# Targeting immunogenic cell death in cancer

**DOI:** 10.1002/1878-0261.12851

**Published:** 2020-12-01

**Authors:** Asma Ahmed, Stephen W.G. Tait

**Affiliations:** ^1^ Cancer Research UK Beatson Institute Glasgow UK; ^2^ Institute of Cancer Sciences University of Glasgow UK

**Keywords:** cancer, caspase, cell death, DAMPs, immunogenic cell death, interferon

## Abstract

Immunogenic cell death (ICD) is a type of cancer cell death triggered by certain chemotherapeutic drugs, oncolytic viruses, physicochemical therapies, photodynamic therapy, and radiotherapy. It involves the activation of the immune system against cancer in immunocompetent hosts. ICD comprises the release of damage‐associated molecular patterns (DAMPs) from dying tumor cells that result in the activation of tumor‐specific immune responses, thus eliciting long‐term efficacy of anticancer drugs by combining direct cancer cell killing and antitumor immunity. Remarkably, subcutaneous injection of dying tumor cells undergoing ICD has been shown to provoke anticancer vaccine effects *in vivo*. DAMPs include the cell surface exposure of calreticulin (CRT) and heat‐shock proteins (HSP70 and HSP90), extracellular release of adenosine triphosphate (ATP), high‐mobility group box‐1 (HMGB1), type I IFNs and members of the IL‐1 cytokine family. In this review, we discuss the cell death modalities connected to ICD, the DAMPs exposed during ICD, and the mechanism by which they activate the immune system. Finally, we discuss the therapeutic potential and challenges of harnessing ICD in cancer immunotherapy.

## Graphical abstract

Immunogenic cell death (ICD) is a form of cell death that can engage immunity. Therapeutic engagement of ICD in cancer may lead to more effective responses by eliciting antitumor immunity. Here, we discuss various modalities of ICD, highlighting our current understanding of the molecular basis of ICD. Finally, we outline the potential and challenge of harnessing ICD in cancer immunotherapy.

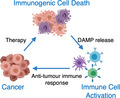

**Abbreviations**
ATP: adenosine triphosphate
BAK: BCL‐2 homologous antagonist killer
BAX: BCL‐2‐associated X protein
BCL‐2: B‐cell lymphoma 2
BID: BH3‐interacting domain death agonist
c‐FLIP: cellular FLICE‐like inhibitory protein
cGAMP: cyclic guanosine monophosphate–adenosine monophosphate
cGAS: cyclic GMP‐AMP synthase
CRT: calreticulin
CXCL10: chemokine C‐X‐C motif ligand 10
DAMPs: damage‐associated molecular patterns
DCs: dendritic cells
DISC: death‐inducing signaling complex
ER: endoplasmic reticulum
FADD: FAS‐associated protein with death domain
FASL: FAS ligand
GSDMD: gasdermin D
GSDMD^NT^: N‐terminal fragment of gasdermin D
GSDME: gasdermin E
HMGB1: high‐mobility group box‐1
HSP: heat‐shock proteins
Hyp‐PDT: hypericin‐based photodynamic therapy
ICD: immunogenic cell death
IFN: interferon
IFNAR: IFN‐α and IFN‐β receptors
IL: interleukin
IRF3: interferon regulatory factor 3
ISGs: IFN‐stimulated genes
LPS: lipopolysaccharide
MAPK: mitogen‐activated protein kinase
MHC: major histocompatibility complex
MLKL: mixed‐lineage kinase‐like
MOMP: mitochondrial outer membrane permeabilization
mtDNA: mitochondrial DNA
NF‐κB: nuclear factor kappa‐light‐chain‐enhancer of activated B cells
NK cells: natural killer cells
NLR: NOD‐like receptor
NLRP3: NOD‐like receptor family, pyrin domain‐containing 3 protein
P2RX7: purinergic receptor P2X 7
PD‐L1: programmed death ligand
PRRs: pattern recognition receptors
PS: phosphatidyl serine
RCD: regulated cell death
RIPK1: receptor‐interacting serine/threonine protein kinase 1
RIPK3: receptor‐interacting serine/threonine protein kinase 3
ROS: reactive oxygen species
STING: stimulator of interferon genes
tBID: truncated form of BID
TBK1: TANK‐binding kinase 1
TLR: Toll‐like receptor
TNF: tumor necrosis factor
TRAIL: TNF‐related apoptosis‐inducing ligand
ZBP: Z‐DNA‐binding protein

## 1 Introduction

Immunogenic cell death (ICD) is a form of regulated cell death (RCD) that is sufficient to activate an adaptive immune response in an immunocompetent setting [1]. It has been classified as a distinct poorly defined entity induced by certain chemotherapeutic drugs, oncolytic viruses, physicochemical therapies, photodynamic therapy, and radiotherapy [1, 2]. Recently, Legrand *et al*. proposed to define ICD on the basis of the communication between dying and immune cells as a successful dialog between a dying cell and an appropriately disposed immune system [3]. Nevertheless, the molecular mechanisms of this interaction are yet to be defined. The long‐standing idea that the immune system functions by merely making a distinction between self and non‐self has been revised with the introduction of the danger model that posits a model in which our immune system responds to entities that cause damage as opposed to those that are simply foreign [4]. Dying or stressed cells release molecules that can function as either adjuvants or danger signals for the immune system. These signals are collectively called damage‐associated molecular patterns (DAMPs) [5]. Importantly, ICD encompasses the release of DAMPs from dying tumor cells, that are recognized by innate pattern recognition receptors (PRRs), such as Toll‐like receptors (TLRs) and NOD‐like receptors (NLRs) resulting in the activation of tumor‐specific immune responses, thus provoking long‐term efficacy of anticancer drugs by combining direct cancer cell killing and antitumor immunity, generally associated with immunological memory [1, 2]. Herein, we discuss different stimuli of ICD, the role of DAMPs in T‐cell cross‐priming, and the activation of antitumor immune response. Finally, we discuss future perspectives for therapeutic targeting of ICD in cancer therapy.

## 2 Immunogenicity of cell death pathways

**Fig. 1:**
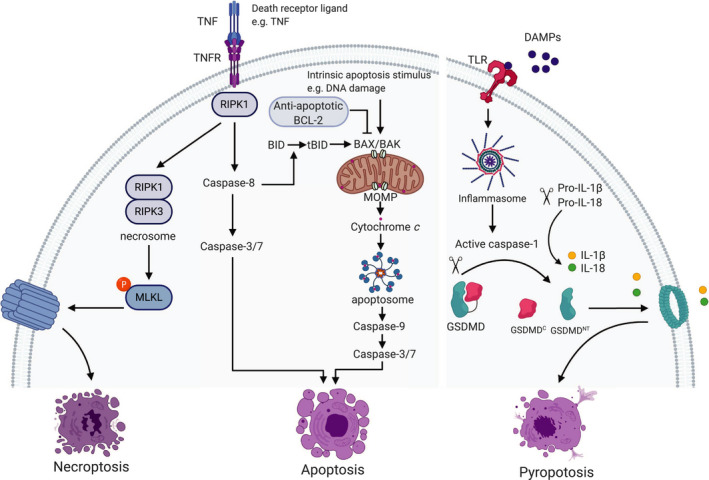
Overview of necroptosis, apoptosis, and pyroptosis signaling pathways. Binding of death ligands such as tumor necrosis factor (TNF) to their cognate receptor (TNFR) leads to pleiotropic signaling including inflammation and cell survival, apoptosis, and necroptosis, determined by the key signaling molecule, the receptor‐interacting serine/threonine protein kinase 1 (RIPK1). Upon inhibition of caspase‐8, RIPK1 activates RIPK3 leading to the formation of the necrosome. The necrosome then phosphorylates and activates mixed‐lineage kinase‐like (MLKL) that causes rapid membrane permeabilization and necroptosis. Activation of caspase‐8 leads to activation of caspase‐3 and caspase‐7 and cell death (extrinsic apoptosis). The intrinsic apoptosis pathway is initiated by perturbation in the internal environment including DNA damage that causes mitochondrial outer membrane permeabilization (MOMP). MOMP is regulated by the interaction between pro‐apoptotic and anti‐apoptotic B‐cell lymphoma 2 (BCL‐2) family proteins. The pro‐apoptotic proteins BCL‐2 associated X (BAX) and BCL‐2 homologous antagonist killer (BAK) form pores at the mitochondrial outer membrane leading to the subsequent release of cytochrome *c* and the formation of the APAF‐1 apoptosome. The apoptosome activates caspase‐9, which subsequently activates caspase‐3 and caspase‐7 resulting in apoptosis. Caspase‐8 mediates the crosstalk between the intrinsic and extrinsic apoptosis pathways by cleaving the pro‐apoptotic BH3‐interacting domain death (BID) to truncated BID (tBID) that activates BAX and BAK. Damage‐associated molecular patterns (DAMPs) released from dying cells activate pattern recognition receptors such as Toll‐like receptors (TLR). This leads to the activation of canonical inflammasomes that activate caspase‐1. Active caspase‐1 cleaves gasdermin D (GSDMD) liberating an N‐terminal (GSDMD^NT^) pore‐forming fragment from the C‐terminal (GSDMD^C^) inhibitory fragment. GSDMD^NT^ form pores leading to membrane permeabilization and pyroptosis. Active caspase‐1 also cleaves the pro‐inflammatory cytokines interleukin 1β (IL‐1β) and IL‐18 into their mature form that are released by GSDMD pores.

Accumulating evidence has shown that dying cells actively regulate immune responses as they release or expose molecules that serve as danger signals to stimulate the innate immune system. Nonetheless, the interaction between cell death trigger and the downstream molecular pathways that determines cell death immunogenicity is highly complex [3, 6]. Here, we discuss the cell death pathways commonly described to induce an immune response, focusing on apoptosis, necroptosis, and pyroptosis (Fig. 1). Other cell death modalities are extensively reviewed elsewhere [1].

### 2.1 Apoptosis

Apoptosis is a form of RCD important for development, tissue homeostasis, and immunity [7]. During apoptosis, cells undergo cytoplasmic shrinkage, nuclear fragmentation, chromatin condensation, and plasma membrane blebbing followed by the formation of apoptotic bodies that are efficiently and rapidly cleared by phagocytes [8, 9, 10]. Apoptosis is mediated by the activity of caspase proteases and can be engaged by two modes: intrinsic and extrinsic, both converge upon activation of caspase‐3 and caspase‐7 (Fig. 1) [11]. Intrinsic apoptosis is triggered by perturbation in the environment involving DNA damage, endoplasmic reticulum (ER) stress, excessive reactive oxygen species (ROS) formation, and replication stress. The key event for intrinsic apoptosis is mitochondrial outer membrane permeabilization (MOMP) [11], that is regulated by the interactions between the pro‐apoptotic and the anti‐apoptotic B‐cell lymphoma 2 (BCL‐2) family members [12]. The pro‐apoptotic proteins BCL‐2‐associated X (BAX) and BCL‐2 homologous antagonist killer (BAK) permeabilize the mitochondrial outer membrane; subsequently, cytochrome *c* and other soluble proteins are released from the mitochondrial intermembrane space resulting in caspase activation and cell death (Fig. 1) [11].

Extrinsic apoptosis is engaged following binding of death ligands including FAS ligand (FASL), tumor necrosis factor (TNF), or TNF‐related apoptosis‐inducing ligand (TRAIL) to their cognate receptors, FAS, TNFRSF1A, and TNFRSF10A and TNFRSF10B receptors, respectively [3]. FAS and TRAIL induce the assembly of the death‐inducing signaling complex (DISC), whereas TNF ligation induces complex I and complex II. These complexes function as a platform to regulate caspase‐8 activation [1]. The DISC is composed of FAS‐associated protein with death domain (FADD), caspase‐8, and cellular FLICE‐like inhibitory protein (c‐FLIP) [3]. In contrast to FAS and TRAIL, the primary signaling output of TNF is not death but rather cell survival via complex I that induces the activation of nuclear factor kappa‐light‐chain‐enhancer of activated B cells (NF‐κB) and mitogen‐activated protein kinase (MAPK). This ultimately leads to the production of inflammatory cytokines and prosurvival proteins such as c‐FLIP. The receptor‐interacting serine/threonine protein kinase 1 (RIPK1) is a key signaling molecule that actively determines the balance between inflammation and cell survival, apoptosis, and necroptosis, a form of caspase‐independent RCD (Fig. 1) [13]. TNF‐induced cell death is tightly regulated by several checkpoints. Upon removal of these brakes, complex II is formed comprising RIPK1, FADD, caspase‐8, and c‐FLIP. Formation of complex II leads to the activation of caspase‐8 that activates caspase‐3 and caspase‐7 and mediates the crosstalk between intrinsic apoptosis and extrinsic apoptosis by cleaving pro‐apoptotic BH3 interacting domain death agonist (BID). The active truncated form of BID (tBID) then activates BAX and BAK and effectively triggers MOMP (Fig. 1) [13].

Although MOMP is critical for intrinsic apoptosis, caspases are not, as typically cells die post‐MOMP in the absence of caspase activity. Caspases appear to function primarily to accelerate cell death—this serves important functions during development and keeps apoptosis immunologically silent [14, 15, 16, 17]. For example, apoptotic caspases cleave and inactivate cyclic GMP‐AMP synthase (cGAS) and interferon regulatory factor 3 (IRF3) to suppress type I interferon (IFN) response [18]. Caspases also inactivate DAMPs indirectly such as high‐mobility group box‐1 (HMGB1) [19]. Thus, engaging MOMP while blocking caspases strongly provokes ICD through the activation of NF‐κB and the induction of mitochondrial DNA (mtDNA)‐mediated type I IFN responses [14, 16, 17]. In line with this, caspase inhibition has been shown to induce antitumor activities accompanied by tumor regression [14]. Furthermore, emricasan, a pan caspase inhibitor, synergizes with radiation and the immune checkpoint inhibitor, anti‐programmed death ligand (PD‐L1), to induce systemic antitumor effects [20].

While most of anticancer therapies induce apoptosis, only a few do so in immunogenic way [21]. Those include anthracyclines [22], oxaliplatin, oncolytic viruses, radiotherapy, and photodynamic therapy [2, 23]. Such therapies are proposed to act by inducing the release of DAMPs in addition to cell death [1]. Remarkably, subcutaneous injection of cancer cells treated with the aforementioned therapies induces a potent cancer vaccine effect [5, 23, 24].

### 2.2 Necroptosis

Necroptosis is a lytic form of RCD that requires the activation of the kinases RIPK1 and RIPK3, which assemble into the necrosome. Necroptosis is initiated by death receptors including TNF, PRRs such as TLR3 that sense viral nucleic acids, TLR4 detecting bacterial lipopolysaccharide (LPS), and the activation of the intracellular sensor Z‐DNA‐binding protein, ZBP (also known as DAI), that senses cytoplasmic viral proteins. Necroptosis downstream TNF signaling requires inhibition of caspase‐8 in complex II resulting in the activation of RIPK3 by RIPK1 [13, 21, 25, 26]. Once activated, RIPK3 phosphorylates mixed‐lineage kinase‐like (MLKL) leading to its oligomerization at the plasma membrane, causing rapid membrane permeabilization (Fig. 1) and the subsequent release of immunogenic DAMPs that robustly activate the innate and adaptive immune systems [25, 27, 28].

In contrast to apoptotic and lytic necrotic cells, necroptotic dying cells mediate DAMP release, increase antigen uptake by phagocytes, and induce dendritic cell (DC) maturation leading to efficient CD8^+^ T‐cell cross‐priming, a functional outcome of antigen cross‐presentation, whereby antigen‐specific naïve CD8^+^ T cells are activated to become cytotoxic T lymphocytes, and antitumor immunity. These effects require RIPK3 and RIPK1‐mediated induction of NF‐κB but not DAMP release [29, 30]. Conversely, another study demonstrated that immunogenicity of necroptotic cells does not correlate with the extent of NF‐κB but rather with DAMP release [27]. Hence, although it becomes apparent that RIPK1 and RIPK3 are required for necroptotic cell immunogenicity, the underlying mechanisms are controversial.

Due to the immunogenic nature of necroptosis, necroptotic cells have been effectively used to induce potent antitumor activity and tumor vaccination [27, 29, 30]. Consistent with this notion, high expression levels of RIPK3 in melanoma patients correlated with improved patient survival [29]. Moreover, RIPK3 expression is lost in many cancer cell lines and in primary human cancers [27]. Recently, it has been shown that oncolytic virus‐mediated induction of RIPK3 expression in tumor cells conferred durable tumor clearance and an abscopal effect [29]. Thus, targeting necroptosis represents an attractive therapeutic approach in cancer therapy especially in apoptosis‐resistant tumors.

### 2.3 Pyroptosis

Pyroptosis is a lytic pro‐inflammatory form of RCD culminating with the formation of plasma membrane pores by members of the gasdermin protein family, primarily gasdermin D (GSDMD), as a consequence of inflammatory caspase activation (Fig. 1). Inflammatory caspases are comprised of caspase‐1, caspase‐4, and caspase‐5 in human, and caspase‐1 and caspase‐11 in mice. Primarily pyroptosis is observed in monocytes and macrophages but can also occur in epithelial cells [31, 32]. Caspase‐1 activation is initiated by ligands of canonical inflammasomes, which are signaling complexes assembled upon detection of host or pathogen‐derived danger signals (Fig. 1). Instead, cytosolic bacterial LPS can directly activate caspase‐4 and caspase‐5 in humans or caspase‐11 in mice [33]. These caspases cleave GSDMD liberating an N‐terminal fragment (GSDMD^NT^), which has intrinsic pore‐forming activity resulting in pyroptosis and the release of DAMPs (Fig. 1) [31, 33]. Caspase‐8 can also cleave GSDMD following apoptotic stimulation [34], whereas GSDME is cleaved by caspase‐3 and caspase‐8 to induce pyroptosis either directly or secondary to apoptosis [31, 35, 36]. Of note, GSDME is silenced in most cancers but expressed in normal tissue. Thus, GSDME‐induced pyroptosis has been proposed to underly the toxicity of chemotherapy drugs [36]. Intriguingly, restoration of GSDME expression in cancer cells suppresses tumor growth mediated by enhancing the phagocytosis of tumor cells by tumor‐associated macrophages and the activation of tumor‐infiltrating natural killer (NK) and CD8^+^ T cells [37]. Moreover, the activation of pyroptosis in tumor cells induces effective antitumor immunity that synergizes with anti‐PD1 immune checkpoint blockade [38].

Interestingly, the GSDME^NT^ can localize to mitochondria and induce MOMP. Likewise, the inflammasome‐generated GSDMD^NT^ can permeabilize mitochondria linking inflammasome activation to apoptosis. These data underscore a role of pyroptosis in inducing MOMP to enhance apoptosis [39]. Active caspase‐1 and caspase‐8 process the pro‐inflammatory cytokines interleukin 1β (IL‐1β) and IL‐18 into their mature form. Once matured, they are released by GSDMD and GSDME pores (Fig. 1) [3, 40]. Of note, the activation of the NLR family, pyrin domain‐containing 3 protein (NLRP3) inflammasome, can cause GSDMD‐mediated IL‐1 β release from living macrophages indicating a nonpyroptotic role of GSDMD [41].

## 3 DAMPS: mediators of immunogenicity

**Fig. 2:**
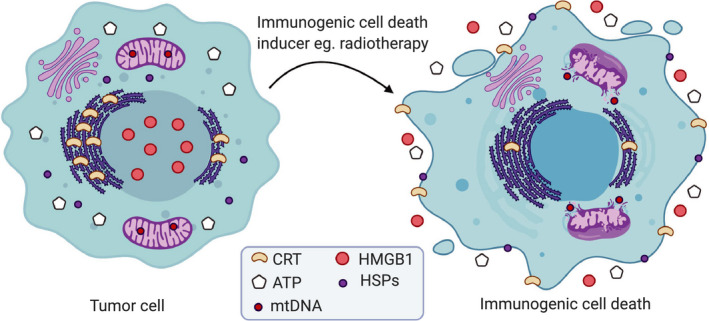
Characteristics of immunogenic cell death. When tumor cells succumb to immunogenic cell death (ICD) for example following radiotherapy, they release or expose damage‐associated molecular patterns (DAMPs) that stimulate antitumor immune responses. Tumor cells expose the endoplasmic reticulum protein calreticulin (CRT) on their plasma membrane in the pre‐apoptotic stage. They might also expose heat‐shock proteins (HSPs). These factors facilitate the uptake of dying cells by phagocytes. Additionally, tumor cells responding to ICD inducer release adenosine triphosphate (ATP) and the nuclear protein high‐mobility group box‐1 (HMGB1) in the extracellular space. Mitochondrial outer membrane permeabilization enables the release of mitochondrial DNA (mtDNA) into the cytosol.

Upon induction of ICD, dying tumor cells release or expose DAMPs (Fig. 2). Yatim *et al*. classified DAMPs as constitutive DAMPs (cDAMPs), which are immune‐stimulatory endogenous molecules that are constitutively expressed before death and are released by dying cells, and inducible DAMPs (iDAMPs), which are the endogenous molecules within dying cells generated during cell death via mechanisms that are dependent on the underlying cell death pathway [6]. DAMPs include nucleic acids such as mtDNA detected by cGAS and stimulator of interferon genes (STING) pathway, the cell surface exposure of ‘eat me’ signals such as calreticulin (CRT) and heat‐shock proteins (HSP70 and HSP90) that promote the cell uptake by APCs, extracellular release of immunostimulatory factors including adenosine triphosphate (ATP), HMGB1, and cytokines such as type I IFNs and IL‐1 family (Fig. 2) [2, 24, 42, 43, 44].

Of note, many ICD inducers induce multiple DAMPs, for example, anthracyclines, radiotherapy, and Hyp‐PDT provoke ICD via inducing the surface exposure of CRT and HSPs, the secretion of ATP and HMGB1, and the release of mtDNA that stimulates the production of type I IFNs (Fig. 2) [2].

### 3.1 Type I IFNs and IL‐1 family cytokines

The rapid production of type I IFNs contributes to the anticancer effects of certain chemotherapeutics [45]. Cancer cells succumbing to ICD produce type I IFNs through the activation of TLR3 by self‐RNA emitted from dying cells [45] or the cGAS/STING pathway in response to mtDNA release [46]. Hence, the degradation of extracellular nucleic acids that result in their reduced sensing by phagocytes such as DCs, neutrophils, and macrophages, considerably limits the immunogenicity of RCD. Type I IFNs mediate broad immunostimulatory effects on the aforementioned immune cells [1]. Additionally, by binding to IFN‐α and IFN‐β receptors (IFNAR I) on cancer cells, type I IFNs trigger autocrine and paracrine signaling pathways culminating in the expression and release of IFN‐stimulated genes (ISGs) including the T‐cell chemoattractant chemokine C‐X‐C motif ligand 10 (CXCL10). Therefore, tumors lacking Tlr3 or Ifnar I failed to respond to chemotherapy [45]. Moreover, a type I IFN‐related signature predicted clinical responses to anthracycline‐based chemotherapy and correlated with improved survival in patients with breast carcinoma characterized by poor prognosis [45, 47].

Members of the IL‐1 cytokine family are powerful iDAMPs. These cytokines interact with surface receptors expressed in wide range of cells, including immune, endothelial, and epithelial cells [44]. All these cell types can produce and secrete IL‐1 cytokines in response to infection, trauma, or NF‐kB activation to stimulate various immune responses. Therefore, the IL‐1 cytokine family appears to act as one of the last signals that alerts the immune system for the presence of dying cells [3]. An accumulating body of evidence suggests that various stimuli that induce inflammatory responses to necrotic cell death act through releasing IL‐1 family members, including ATP. Administration of purified IL‐1 family cytokines *in vivo* is capable of promoting the synthesis and secretion of numerous additional cytokines and chemokines to promote phagocyte recruitment and the production of inflammatory responses [44]. In line with this notion, neutralizing antibodies to IL‐1 receptor or IL‐1β blunt the therapeutic response of tumors to chemotherapy *in vivo* by reducing T‐cell priming [43]. Consistently, IL‐1α has been shown to enhance the antitumor efficacy of the epidermal growth factor inhibitor, cetuximab, in head and neck squamous cell carcinoma patients [48].

### 3.2 Calreticulin

Calreticulin (CRT) is a soluble protein in the lumen of the ER where it mediates several functions. Those include chaperone activity and the regulation of calcium homeostasis and signaling, proper assembly of major histocompatibility complex (MHC) class I molecules, and the loading of antigen. Surface exposure of CRT is an important ‘eat me’ signal that interacts with CD91 receptors in phagocytes and enables them to efficiently engulf dead cells [5, 49]. In the course of ICD, CRT translocation from the ER occurs before phosphatidyl serine (PS) exposure. This early, pre‐apoptotic exposure of CRT on the plasma membrane facilitates the uptake of dying cells by DCs, which lead to subsequent tumor antigen cross‐presentation and tumor‐specific cytotoxic T lymphocyte responses [24]. Consistent with a role of CRT in mediating immunogenicity of ICD, knockdown of CRT or defects in CRT exposure pathway abolishes the ability of dying cancer cells succumbing to anthracyclines to establish protective immunity in mice, while the provision of exogenous CRT confers immunogenicity of otherwise nonimmunogenic types of RCD. Accordingly, CRT exposure has been shown to have a positive prognostic value in patients with acute myeloid leukemia [1].

### 3.3 ATP

Extracellular ATP released from dying cells acts as a ‘find me’ signal and is required for the generation of robust chemotherapy‐induced antitumor immune responses. Consequently, ATP depletion abolishes the immunogenicity of cell death [1, 5]. Following cell death, ATP release can occur secondary to caspase‐3‐ or caspase‐7‐mediated activity. Additionally, the optimal release of ATP from dying cells has been shown to require autophagy. Consequently, genetic or pharmacological inhibition of autophagy strongly reduces ATP secretion from dying cancer cells and dampens their immunogenicity [49]. Following chemotherapy, ATP released is sensed by purinergic P2RX7 receptors on DCs and induces their recruitment. ATP also mediates immunostimulatory effects by activating the NLRP3 inflammasome and the subsequent secretion of IL‐1β. This step is mandatory for the DC‐mediated immunogenicity of cell death. Hence, oxaliplatin‐treated tumor cells failed to prime T cells for IFN‐γ production when they were inoculated into P2rx7 receptor‐deficient hosts. Furthermore, anthracycline‐treated breast cancer patients carrying a loss‐of‐function allele of P2RX7 developed metastatic disease more rapidly than individuals carrying the normal allele [43].

### 3.4 HMGB1

HMGB1 is an abundant nuclear nonhistone chromatin‐binding protein. An important characteristic of ICD is the release of HMGB1 from the nucleus into the surroundings of dying cells (Fig. 2). HMGB1 mediates potent pro‐inflammatory effects by binding to TLR4 on DCs to stimulate efficient processing and cross‐presentation of tumor antigens from dying cells [43, 50]. In a tumor vaccination mouse model, using HMGB1‐depleted tumor cells or neutralizing HMGB1 by specific antibodies compromised the ability of mice to resist tumorigenesis. Furthermore, breast cancer patients with a TLR4 loss‐of‐function allele, that prevents HMGB1 binding to TLR4, are more prone to relapse after radiotherapy or chemotherapy [50]. Besides, another study indicated that HMGB1 synergizes with ATP to induce IL‐1β release by DCs and that antibody specific for HMGB1 abrogated the ability of DCs to produce IL‐1β in contact with dying tumor cells [43]. Taken altogether, these observations delineate an important role of HMGB1 for cell death immunogenicity. However, accumulating evidence shows that the immunogenic properties of HMGB1 are dependent upon its redox state. This has been shown by the potent pro‐inflammatory activities of HMGB1‐reduced form in contrast to the inactive oxidized form [51]. Thereby, caspase‐dependent production of ROS that triggers the oxidation of HMGB1 has been shown to dampen its immunostimulatory activity leading to the induction of immunological tolerance of dying cells [19]. In marked contrast, one study has shown that HMGB1 interacted with tumor‐infiltrating DCs to suppress nucleic acid‐mediated antitumor activity in mice with established tumors [52]. Nevertheless, since this immunosuppressive effect of HMGB1 is observed in established tumors where the tumor environment is likely to contain high levels of ROS [53], it could be that the inactive oxidized form of HMGB1 is responsible for the observed tolerogenic effects in this scenario.

### 3.5 HSPs

Intracellular HSPs are highly conserved proteins that have strong cytoprotective and anti‐apoptotic properties. They behave as molecular chaperones for other cellular proteins [54]. One of the first reports that described the role of immunogenicity of cell death in the efficacy of anticancer therapy emerged from the observation that induction of HSP expression in response to cell death increased tumor cell immunogenicity and conferred enhanced clearance of tumors *in vivo* [55]. Subsequent studies demonstrated the cell surface exposure of HSP70 following Hyp‐PDT [23] and HSP90 after chemotherapy leading to an anticancer immune response [56]. Thus, blocking HSP90 abolished chemotherapy‐mediated antitumoral vaccination [56]. Furthermore, the chemotherapy‐induced increased surface expression of HSP70 and HSP90 in dying tumor cells correlated with DC activation. However, this DC activation induced by chemotherapy was completely abolished upon pretreatment of these tumor cells with neutralizing antibodies against the HSP70 and HSP90 and CRT common receptor, CD91 [57].

## 4 Endoplasmic reticulum (ER) stress and ICD induction

A proposed shared characteristic of ICD inducers is their ability to induce ER stress and ROS production [2]. Hence, the more focused the ER stress is, the higher the immunogenicity of cell death is. On this basis, ICD inducers have been classified into type I ICD inducers that prompt secondary or collateral ER stress such as anthracyclines and radiotherapy and type II ICD inducers that foster focused ROS‐based ER stress including hypericin‐based photodynamic therapy (Hyp‐PDT) and oncolytic viruses, with the latter being more immunogenic [2, 5]. The first systematic screening for ICD inducers identified the anthracycline doxorubicin as efficient inducer through its ability to induce ER stress [24]. Supporting this notion, concomitant induction of ER stress restores the immunogenicity of poor ICD inducer chemotherapeutics such as cisplatin [58], etoposide, and mitomycin C [24]. The importance of ROS was evident by the diminished immunogenicity of ICD in the presence of antioxidants [5]. Importantly, ROS‐mediated ER stress induced by Hyp‐PDT has been shown to induce the emission of multiple DAMPs ultimately leading to protective antitumor immune responses [42]. This is because hypericin is an ER‐localizing drug that causes massive production of ROS at the ER when excited by light of a specific wavelength leading to focused ROS‐based ER stress [5].

## 5 T‐cell priming and antitumor immune responses following ICD

**Fig. 3:**
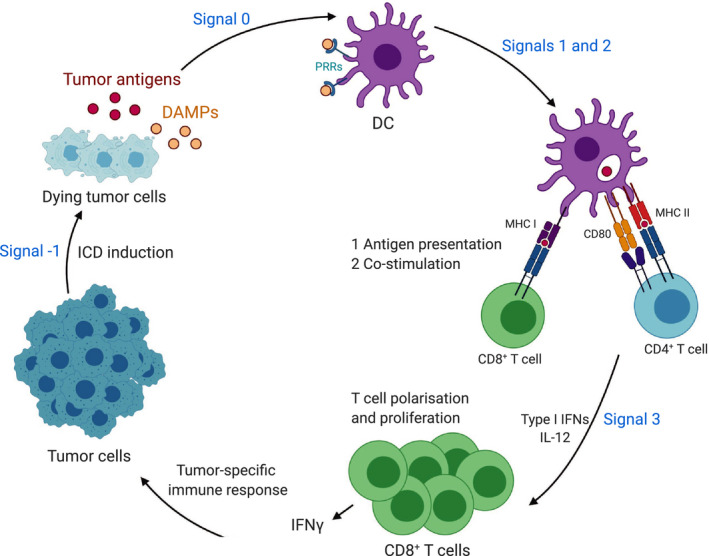
Role of DAMPs in antigen cross‐priming and antitumor immune responses. Upon immunogenic cell death (ICD) induction, dying tumor cells release tumor antigen and damage‐associated molecular patterns (DAMPs). DAMPs stimulate pattern recognition receptors (PRRs) on dendritic cells (DCs) leading to T‐cell activation and the initiation of tumor‐specific antitumor immune responses following five signals (signals −1 to 3). Signal −1 is the cell death process itself that results in DAMPs and provides tumor antigens for antigen cross‐priming. DAMPs mediate the attraction and activation of DCs that engulf and process dying cells (signal 0). DCs provide 3 signals to activate T cells; signal 1 is the antigen recognition mediated through the T‐cell receptor (TCR), trigged by DCs cross‐presentation of tumor antigens on major histocompatibility complex (MHC) I or MHC II molecules to CD8^+^ T cells and CD4^+^ T cells, respectively. Signal 2 is the engagement of naive T cells with co‐stimulatory receptors on DCs such as CD80 which is required for efficient T‐cell activation. Signal 3 is the additional polarization and differentiation signals delivered from DCs, including interleukin 12 (IL‐12) or type I interferons (IFNs), that are crucial for T‐cell differentiation into tumor‐specific IFN‐γ‐producing T cells.

The innate immune system plays a crucial role in the activation of adaptive immunity leading to tumor‐specific immune responses. Consistent with this, harnessing innate immunity has proven to be highly effective in controlling tumor growth and promoting responses to immune checkpoint inhibitors, that unleash effector T cells, in a variety of preclinical and clinical studies (reviewed in ref. [59]). Following ICD, DAMPs stimulate PRRs on macrophages, DCs, and NK cells leading to T‐cell activation and the initiation of immune responses [6]. In order for cell death to provoke an immune response, the dying cell needs to trigger a total of five steps (Fig. 3). The cell death event itself is considered signal −1, while the release of DAMPs and their recognition by PRRs constitutes signal 0 [3, 6]. DAMPs mediate the attraction of phagocytes that engulf and process dying cells to present their antigens on the surface of antigen‐presenting cells such as DCs (Fig. 3). Activated DCs migrate to draining lymphoid organs where they maturate and encounter naive T cells [3, 6, 60]. Signal 1 comprises antigen recognition mediated through the T‐cell receptor and trigged by DCs cross‐presentation of tumor antigens on MHC I or MHC II molecules to CD8^+^ T cells and CD4^+^ T cells, respectively (Fig. 3). Efficient T‐cell activation requires the engagement of naive T cells with co‐stimulatory receptors on DCs such as CD80 and CD86 (signal 2). Signal 3 is the additional polarization and differentiation signals delivered from DCs, including IL‐12 or type I IFNs, that are crucial for T‐cell differentiation into a T‐cell effector. All these events eventually lead to priming of tumor‐specific CD8^+^ T cells (Fig. 3) [3, 6, 59].

Noteworthy, although DAMPs such as ATP and HMGB1 induce DC maturation and provoke cytokine release, they are insufficient for the induction of effective T‐cell cross‐priming highlighting the need for further signals [30]. Phagocytes are attracted by ‘find me’ signals that are released by dying cells including ATP and NF‐κB‐induced signals that trigger phagocyte infiltration. Once phagocytes reach the site of tissue damage, they recognize ‘eat me’ signals on the surface of dying cells such as PS. In contrast, the surface of healthy cells displays ‘don’t eat me’ signals, such as CD47 [6]. Consistently, increased CD47 expression on tumor cells prevents DNA sensing in DCs leading to immune evasion. Thus, the inhibition of CD47 enhances tumor antigen sensing by DCs eliciting effective T‐cell priming and tumor rejection in mice [60].

As discussed earlier, the therapeutic efficacy of anticancer chemotherapies largely depends on DCs, which present antigens from dying cancer cells to prime tumor‐specific IFN‐γ‐producing T lymphocytes (Fig. 3) [43, 60]. Dying tumor cells release ATP, which activates the NLRP3 inflammasome in DCs allowing for the secretion of IL‐1β, a cytokine which is required for the polarization of IFN‐γ‐producing CD8^+^ T cells. Therefore, the priming of IFN‐γ‐producing CD8^+^ T cells by dying tumor cells fails in the absence of a functional IL‐1 receptor 1 and in Nlrp3‐deficient or caspase‐1‐deficient mice unless exogenous IL‐1β is provided. Consistent with this finding, tumors established in Nlrp3‐deficient or caspase‐1‐deficient hosts were not effectively targeted by chemotherapy [43]. In summary, robust antitumor immune responses require the efficient priming of tumor antigen‐specific IFN‐γ‐producing CD8^+^ T cells that mediate tumor clearance.

## 6 Inflammatory pathways activated during ICD

The NF‐κB pathway regulates diverse cellular processes including proliferation and cell survival, cytokine production, antipathogen response, and inflammation. NF‐κB is tightly connected to decisions of life and death, particularly during extrinsic apoptosis and necroptosis [3, 13]. In the context of ICD, uncoupling cell death from NF‐κB activation dampens the pro‐inflammatory response elicited by dying cells and leads to inefficient CD8^+^ T‐cell priming [14, 30]. Supporting this notion, robust CD8^+^ T‐cell cross‐priming has been shown to critically require active RIPK1 and NF‐κB signaling within dying cells, but not the DAMPs released by these cells [30]. Nonetheless, how NF‐κB activation within dying cells actively regulates cross‐priming is unknown [6].

**Fig. 4:**
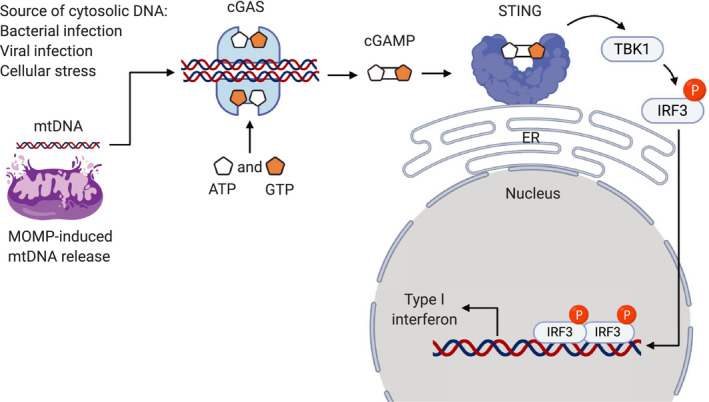
cGAS‐STING signaling pathway. The presence of cytosolic DNA either through bacterial or viral infections or cellular damage leading to mitochondrial outer membrane permeabilization (MOMP)‐induced mitochondrial DNA (mtDNA) release activates cyclic GMP‐AMP synthase (cGAS). Activated cGAS forms a dimeric cGAS‐DNA complex, which synthesizes cyclic guanosine monophosphate–adenosine monophosphate (cGAMP) from ATP and GTP. cGAMP binds to and activates the endoplasmic reticulum (ER) membrane protein, stimulator of interferon genes (STING), that subsequently recruits TANK‐binding kinase 1 (TBK1). TBK1 phosphorylates and activates the transcription factor interferon regulatory factor 3 (IRF3) leading to the production of type I interferons.

The presence of cytosolic DNA either through bacterial or viral infections or cellular damage triggers robust pro‐inflammatory immune responses [61]. Notably following MOMP, the release of mtDNA into the cytosol activates a plethora of innate immune responses, including cGAS‐STING that triggers type I IFN responses (Fig. 4), TLR9 which activates NF‐κB, and inflammasome formation leading to pyroptosis and the maturation of IL‐1β and IL‐18 (reviewed in ref. [46]). Cytosolic DNA activates cGAS to form a dimeric cGAS‐DNA complex, which synthesizes cyclic guanosine monophosphate–adenosine monophosphate (cGAMP) from ATP and GTP (Fig. 4). cGAMP in turn binds and activates the ER membrane protein STING that subsequently recruits TANK‐binding kinase 1 (TBK1). TBK1 phosphorylates and activates the transcription factors IRF3 and NF‐κB, leading to the production of type I IFNs and other cytokines (Fig. 4) [46, 61]. Accumulating evidence demonstrated that apoptotic caspase activity dampens the production of mtDNA‐induced type I IFNs [16, 17, 18], implicating a therapeutic potential for caspase inhibition in cancer therapy. Supporting this idea, engaging MOMP under caspase‐inhibited conditions results in activation of NF‐κB‐mediated upregulation of cytokines and chemokines that promotes activation of antitumorigenic macrophage and cytotoxic CD8^+^ T cells leading to robust antitumor immune responses culminating in tumor regression [14]. Likewise, a recent study showed that concomitant treatment of mice with caspase inhibitor and radiation results in robust antitumor immune responses as a consequence of enhanced tumor‐derived mtDNA‐mediated production of type I IFNs [20].

Type I IFNs have long been used for the effective treatment of multiple cancers even before their mechanism of action was known and proposed to be utilized as adjuvant to cancer vaccines [62]. The importance of type I IFNs in mediating antitumor immune responses is evidenced by the fact that blocking type I IFNs and the deficiency of IFNAR I abrogates tumor rejection [63]. Type I IFNs stimulate the activation of DCs following engulfment of apoptotic tumor cells, sustain tumor antigen‐bearing DCs survival, and enhance antigen cross‐presentation leading to CD8^+^ T‐cell cross‐priming [64]. The rapid induction of type I IFNs in tumor cells underlines the mechanism of ICD following anthracycline treatment of multiple cancer cell lines. This effect required signaling via IFNAR I for efficient antitumor immunity [45]. Similarly, the cGAS‐STING‐IRF3‐type I IFN cascade mediates a robust antitumor effects following radiation [65]. Thus, enhancing cGAS‐STING signaling leading to type I IFN production represents a promising therapeutic approach for cancer treatment.

## 7 Conclusions and perspectives

The concept of cancer immunotherapy is to harness the immune system to elicit antitumor immune response. Accumulating evidence clearly underscored the ability of DAMPs released from dying cells to activate specific antitumor immune responses. Understanding the molecular pathways by which certain cancer therapeutics induce DAMPs and activate the host immune system offer the potential to convert nonimmunogenic inducers of cell death to potent ICD inducers and open the prospect for improving tumor vaccination strategy. Despite tremendous efforts aiming to characterize ICD, the current knowledge is far from being established. Outstanding questions exist: What makes a certain cell death modality immunogenic? Similarly, what are the factors affecting the immunogenicity of a dying cell? Additionally, since most of the ICD research is conducted in cancer cells and tumor vaccination models, it would be of utmost importance to test ICD in more advanced tumor models such as orthotopic and genetically engineered mouse models to reflect the complexity of human disease. Noteworthy, although ICD has been shown in several preclinical models, evidence for ICD in human patients is less compelling. Therefore, further research in human patients is needed to investigate the potential of ICD in clinical settings. Notably, identifying biomarkers that stratify patients according to their benefit from ICD immunotherapy would be of great advantage. Furthermore, it is tempting to speculate that the administration of single or multiple ICD inducers, in addition to harnessing innate immunity and/or immune checkpoint inhibition, is likely to boost the antitumor immune responses resulting in abscopal effect conferring long‐term systemic protection from cancer development. Nevertheless, these assumptions remain to be tested as the ICD field is actively expanding and special consideration of the dosage and treatment regiments must be kept in mind to avoid toxicities and the emergence of resistance.

## Conflict of interest

The authors declare no conflict of interest.

## Author contributions

AA and SWGT conceived and designed the manuscript. AA wrote the text and prepared the figures, and SWGT edited the manuscript.

### Peer Review

The peer review history for this article is available at https://publons.com/publon/10.1002/1878‐0261.12851.
